# An illustrated booklet for reinforcing community health worker knowledge of tuberculosis and facilitating patient counselling

**DOI:** 10.4102/phcfm.v10i1.1687

**Published:** 2018-05-24

**Authors:** Ida L.A. Okeyo, Ros Dowse

**Affiliations:** 1 Faculty of Pharmacy, Rhodes University, South Africa; 2 School of Public Health, University of Western Cape, South Africa

## Abstract

**Background:**

Community health workers (CHWs) have facilitated the move to decentralise tuberculosis (TB) management, but lack access to information appropriate both for personal use and in patient interaction and education.

**Aim:**

To explore the impact of a pictorial-based TB booklet on reinforcing CHW knowledge and facilitating patient counselling.

**Setting:**

This study was conducted in local primary health care clinics and the Hospice in Grahamstown, Eastern Cape Province in South Africa.

**Methods:**

Quantitative and qualitative methods were used. A simple, 17-page, A5 booklet containing pictograms and simple text was designed in collaboration with CHWs who advised on preferred content. Its influence on knowledge was assessed in 31 CHWs using a 17-item questionnaire in a before-and-after study. The experiences of CHWs using the booklet were qualitatively explored using focus group discussions (FGD) and semi-structured interviews.

**Results:**

Overall knowledge increased significantly from 70.6% to 85.3% (*p* < 0.001) with 8 of 17 questions significantly better answered at follow-up. These addressed meaning of side effects and side effect advice for patients, cause and prevention of TB, action if a dose is forgotten, timing of dose in relation to food intake and the possibility that not all patients are cured. Community health workers reported using the booklet during patient interactions, commenting that it enhanced their confidence in their own TB-related knowledge, improved recall of information and reduced uncertainty. They appreciated the simplicity of the text and its user-friendliness because of the inclusion of pictograms. The booklet was perceived to be valuable as a tool for both patient education as well as improved communication with patients.

**Conclusion:**

A simple, user-friendly TB booklet containing pictograms improved CHW knowledge and acted as a valuable tool in patient communication and education.

## Introduction

Tuberculosis (TB) is a major public health concern with an estimate of 6.3 million new TB cases and 1.7 million people who died from the disease at the end of 2016.^1^ South Africa features as one of the 20 high-burden countries identified by the World Health Organization (WHO) as being responsible for about 90% of the TB global burden.^1^ Despite TB being curable, the disease was responsible for 25 000 deaths in South Africa in 2015 largely fuelled by human immunodeficiency virus (HIV) and/or acquired immunodeficiency syndrome (AIDS) and a rise in drug-resistant TB.^2^ The Directly Observed Therapy, Short Course strategy advocated by the WHO in 1994 was one of the earliest global efforts to reduce TB by ensuring adherence through observation of patients on therapy.^3^ This strategy was incorporated into the South African National TB Programme^4^ and has since been decentralised from hospital and primary health care centres to communities, largely because of a lack of financial and human resources,^5^ a move facilitated through involving community health workers (CHWs).

Evidence of the potential of CHWs has been shown both globally and in South Africa, particularly their role in providing palliative care in HIV and AIDS programmes, acting as adherence supporters, linking communities with the health care system and contributing to social development of their local communities.^6,7,8^ The South African National TB Programme allows for patients taking TB medication to be supervised by CHWs at home,^9^ positioning CHWs as often the only health care personnel with whom patients have frequent contact. This transfers greater responsibility to the CHW to ensure adequate patient education and counselling. Being drawn from the community they serve and sharing the same culture and language, CHWs are at an advantage when communicating with patients, and this promotes trust, positively influences CHW–patient communication and may enhance patient satisfaction and adherence.^10,11^ Despite this realisation, the potential benefit of CHWs within many health care settings, including South Africa, is hampered by challenges such as remuneration of CHWs, inadequate training and supervision, poor consolidation of services and role clarification.^12,13,14^

Knowledge of the disease and its treatment and good communication skills are key components in ensuring quality patient education and communication,^15,16^ with written information playing a key role in contributing to the value of this unique intermediary position inhabited by CHWs.^17,18,19^ Written information, enhanced by visual aids such as pictograms, can enhance comprehension and recall of information.^20,21,22^ When combined with supervision and training, pictorial-enhanced written information materials have been used to improve the practice of CHWs and minimise dependence on memory.^17,18,19,23,24^ Illustrated information may also strengthen patient communication by guiding CHWs in consistently offering accurate information, which promotes adherence with guidelines and reduces guesswork.^19^

A number of reviews that identify features of successful CHW programmes advocate for ongoing training and support for CHWs, which ensures that their information needs are met; however, there is limited evidence of how this can be operationalised in the current settings.^6,8,12,14^ Pakenham-Walsh and Bukachi^25^ in their literature review of information needs of health workers in developing countries highlight that simply producing written materials for health workers is not sufficient. They highlight that there is a need to improve the usability of written materials such as guidelines to ensure they are clear, easy to use, visually attractive and in the appropriate language. Additionally, the review emphasises that there should be a better understanding of local needs prior to the design of materials, which can be achieved by ensuring active participation of health workers.^25^

Informed by an earlier phase of this research which identified unmet information needs of local CHWs for TB information materials,^26^ this study aimed to develop a simple, illustrated TB booklet for CHWs, to evaluate its impact on reinforcing TB knowledge and to explore CHW perceptions of using it in practice.

## Method

### Study site and population

The rural Eastern Cape, where 57% live in poverty,^27^ has the third highest provincial incidence of TB-related deaths.^28^ Initial meetings with the District Health Office and Grahamstown Hospice were followed by site visits by the principal investigator (I.L.A.O.) to introduce the study and collect names of CHWs. All CHWs working with patients with TB were recruited by convenience sampling from Grahamstown’s six primary health care clinics (PHCs) and Grahamstown Hospice. Community health workers could either be TB-specific or generalist, but had to be involved in routine care of patients with TB. All 31 CHWs who were approached agreed to participate and signed consent forms.

### Study design and questionnaire

A before-and-after quantitative knowledge assessment was conducted followed by qualitative interviews and discussions,^29^ which were chosen to complement the knowledge assessment results by gaining further insight into CHW experiences with perceptions of using the booklet. The development of the quantitative questionnaire was informed by previous studies assessing TB knowledge in different groups: health care professionals (HCPs), patients and community members.^30,31,32,33^ The questionnaire was piloted and revised.

### Baseline knowledge assessment

All 31 CHW interviews took place at PHC facilities or at Hospice. They were conducted in English by the principal investigator with an interpreter present to assist in translation to isiXhosa if required. Data collected during the individual CHW interviews included participant characteristics and TB knowledge in the following areas: TB disease, treatment, side effects, and multidrug-resistant (MDR) and extensively drug-resistant (XDR) TB.

### Booklet design and distribution

Design of the booklet was informed by earlier project findings, which identified unmet information needs of CHWs enrolled in this study^26^ and South African TB management guidelines.^9^ Topics included TB prevalence, cause, prevention, people at risk, signs and symptoms, diagnosis, treatment, medicine side effects, MDR and XDR-TB, HIV and/or AIDS and TB, and support for patients with TB.

**FIGURE 1:**
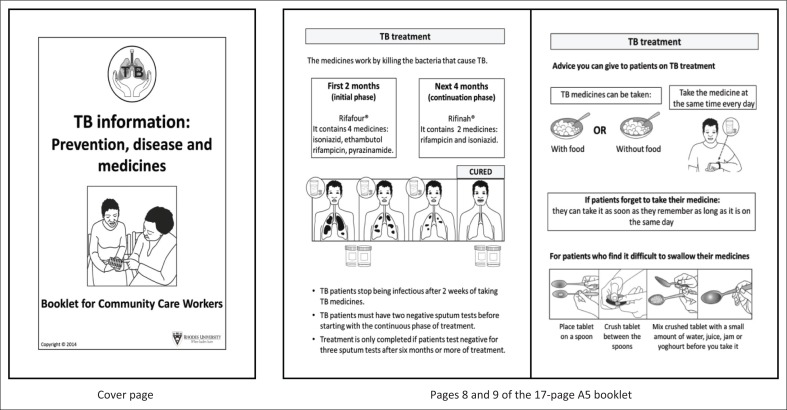
Cover page and two sample inside pages of the booklet on tuberculosis.

To enhance end-user readability, good information design principles were adopted.^34^ An established pictogram design process^35^ was employed to design 26 new pictograms, with 17 existing pictograms being included.^35^ Early booklet versions were modified in discussion with CHWs. Text was minimised, use of medical jargon avoided and text and pictograms juxtaposed to enhance comprehension (Figure 1). The booklet was A5 size, consisted of 17 pages, used a large font size for easy readability. It was translated into isiXhosa and Afrikaans using professional translators and back translated by a different translator.

Three weeks after the baseline knowledge assessment, information sessions were held with all CHWs in each facility during which the principle investigator systematically worked through the final booklet and actively encouraged CHWs to use it in their daily practice for the subsequent 3-month period.

### Follow-up knowledge assessment and exploration of community health workers’ perceptions of booklet

After 3 months, knowledge was re-assessed in all 31 CHWs using the same questionnaire. This was followed by three focus group discussions (FGDs with three to five CHWs) and four individual semi-structured interviews^36^ conducted by the principal investigator in a closed room in each of the seven facilities. The selection of whether to use a focus group discussion versus a semi-structured interview was determined by the number of CHWs who were present at the clinic during the time of interview. Discussions in the focus group and semi-structured interviews followed the same general procedure. Questions were asked by the principal investigator using a question guide and an observer was present to take field notes. Sessions began with trying to establish the extent to which the CHWs had used the booklet in their interactions with patients. The discussions then covered perceptions of the booklet, feedback on its design, patient reaction to the booklet and the overall impression of the CHWs relating to the research study. Each session lasted between 30 and 45 min and was audiotaped with permission from CHWs. Sessions continued until data saturation was achieved and involved 19 CHWs.

### Data analysis

Knowledge scores were compared using the paired *T*-test. The association of selected variables (age, education, years as CHW, TB workshop attendance) with knowledge scores was investigated using Pearson chi-square tests. Statistical significance was set at 5%. Discussions were transcribed verbatim and data analysed thematically.^37^ After reading the transcripts, codes were identified based on key words and recurrent issues that emerged from the data. Coding was done independently by the two researchers who later met to discuss any disparities. Themes were identified from the coded data and further refined, aided by NVivo 10^®^.

### Ethical considerations

Ethical approval was obtained from the Rhodes University Pharmacy Ethics Committee (PHARM 2014 – 10) and the Eastern Cape Department of Health.

## Results

### Demographics and knowledge results

Only 3 of 31 CHWs were men and most participants had been working as CHWs for more than 4 years (Table 1). All could understand English and had isiXhosa as their first language, although 24 of 31 preferred to read the English booklet.

**TABLE 1:** Characteristics of community health workers (*n* = 31).

CHW characteristics	Total *n* (%)	Mean ± s.d.
**Gender**	-	-
Male	3 (9.7)	-
Female	28 (90.3)	-
**Age (years)**	-	38 ± 7.71
≤ 35	14 (45.2)	-
≥ 36	17 (54.8)	-
**Schooling (years)**	-	11 ± 0.79
10–11	15 (48.4)	-
12	16 (51.6)	-
**Type of CHW**	-	-
Lay counsellor	4 (12.9)	-
Directly Observed Therapy supporter	8 (25.8)	-
Home-based carer	13 (41.9)	-
General care worker	6 (19.4)	-
**Time as CHW (years)**	-	-
0–3	8 (25.8)	-
≥ 4	23 (74.2)	-
**Language preference in reading booklet**	-	-
IsiXhosa	6 (19.4)	-
English	24 (77.4)	-
Afrikaans	1 (3.2)	-
**TB workshop in last 12 months**	-	-
Yes	16 (51.6)	-
No	15 (48.4)	-

CHW, community health worker; s.d., standard deviation; TB, tuberculosis.

### Knowledge at baseline and follow-up

From Table 2, mean baseline knowledge was 70.6%, which increased significantly at follow-up to 85.3% (*p* < 0.001). Significant increases in knowledge at follow-up were observed for 8 of 17 questions. All CHWs knew the treatment duration of uncomplicated TB; however, knowledge regarding patients who may not get cured improved at follow-up. Follow-up knowledge of what patients should tell HCPs if taking other medicines remained poor (25.8%), while the timing of TB medication in relation to food intake was significantly (*p* = 0.012) better answered at follow-up (81.6% vs. 61.3%).

**TABLE 2:** Correct responses to individual knowledge questions (*n* = 31).

Questions	Baseline *n* (%)	Follow-up *n* (%)	*p*-value
**Treatment**			
Not all patients with TB get cured	17 (54.8)	27 (87.1)	0.001*
Duration of treatment for TB	31 (100.0)	31 (100.0)	-
Advice for taking TB medicines and other meds	5 (16.1)	8 (25.8)	-
Taking TB medicines with or without food	19 (61.3)	25 (80.6)	0.012*
Avoiding alcohol and smoking while on TB meds	26 (83.9)	27 (87.1)	-
Advice for patients with difficulty swallowing tablets	28 (90.3)	30 (96.8)	-
Action if a dose is forgotten	22 (71.0)	29 (93.5)	0.017*
**TB, MDR-TB and XDR-TB**			
Cause of TB	17 (54.8)	29 (93.5)	< 0.001*
Prevention of TB	22 (71.0)	30 (96.8)	0.003*
Cause of MDR- or XDR-TB	28 (90.3)	30 (96.8)	-
MDR-TB is curable	30 (96.8)	30 (96.8)	-
XDR-TB is curable	25 (80.6)	25 (80.6)	-
Same treatment for TB and drug-resistant TB	23 (74.2)	30 (96.8)	0.006*
Treatment duration for MDR- or XDR-TB	17 (54.8)	23 (74.2)	-
**Side effects**			
Meaning of side effects	19 (61.3)	26 (83.9)	0.006*
Side effects of TB medicines	15 (48.4)	19 (61.3)	-
Advice for patients experiencing side effects	25 (80.6)	31 (100.0)	0.012*
Overall % knowledge score	(76.1)	(85.4)	< 0.001*
Overall knowledge score (mean ± standard deviation)	11.9 ± 2.0	14.5 ± 1.3	-

TB, tuberculosis; MDR, multidrug-resistant; XDR, extensively drug-resistant

* , significant difference (*p* < 0.05) between baseline and follow-up.

Disease-related knowledge pertaining to the cause of TB and its prevention improved significantly after exposure to the booklet. Although CHWs requested information regarding drug-resistant TB,^26^ most were aware of its cause. Booklet information regarding the curability of drug-resistant TB did not alter CHW knowledge score, but did improve the successful explanation of the term ‘side effect’ and the advice that could be offered to patients. However, only 15 of 31 were able to name at least three side effects caused by TB medicines at baseline, which improved to 19 of 31 at follow-up.

The knowledge score was not significantly influenced by age, education, attendance of recent training courses, or length of time practising as a CHW.

### Qualitative: community health workers’ perception of the usefulness of the booklet

The themes that emerged from the semi-structured interviews and the focus group discussions were similar and have therefore been presented together. The four themes that emerged include use of the booklet in practice, role of the booklet in interactions with patients, CHW opinion of the booklet, suggested broader application for similar information materials.

### Use of the booklet in practice

Most CHWs reported actively using the booklet, although a small minority reported no impact of the booklet on their knowledge or practice. The booklet was used in both clinic settings and home visits in different ways: to verify information and answer questions or using the information during counselling, especially for patients recently affected with TB. Many CHWs preferred showing the booklet to patients they were educating especially during home visits or support groups where everyone would have a chance to glance at the booklet. They also found the booklet to be extremely useful in serving as a screening tool for recalling (pictorial) signs and symptoms of TB and referring those in need of testing to the clinic. The pictograms available in the booklet were also mentioned to be a good tool to facilitate memory recall of TB information, which assisted CHWs in their practice.

‘The first time I took it and read, I was so interested, then I show the clients here in front [*waiting area*] what I have and I educate them about TB.’ (Participant 19, female, SSI 4)

‘I use this book in a children support group… It’s easy now to see the signs [*signs of TB*], like if I’ve noticed that the child is coughing a lot and losing weight.’ (Participant 16, female, FGD 3)

‘I meet … adults in the household so I will get together with them maybe in the front room or kitchen and then talk to them like that and then show them the booklet I have and they will understand things better.’ (Participant 12, female, FGD 3)

Because CHWs are required to give daily health education talks at the clinic, they reported that patients often complained of being tired of listening to health talks when they were feeling ill. Community health workers therefore appreciated the booklet, which could be handed to patients to read at their own pace. It also relieved them of the pressure of talking for a long period of time and allowed them more time to spend on other tasks.

‘I gave them while they were waiting [*rather*] than talk and talk … they read and read. Then when I have the time I go there and ask some questions.’ (Participant 19, female, SSI 4)

### Role of the booklet in interactions with patients

Community health workers acknowledged that the illustrated booklet with its attractive visuals facilitated memory recall and made the educational process more meaningful, as patients displayed heightened interest and eagerness to listen, resulting in improved CHW–patient interaction.

‘It actually shows pictures … so with the pictures and us explaining to them the actual way of doing it, it’s very easy to interact with people.’ (Participant 4, male, FGD 1)

‘It helped because I like the pictures, I read then I see in the pictures and I remember.’ (Participant 17, female, SSI 2)

Lack of patient trust in verbal information was mentioned by CHWs, with patients appearing to have better trust in written information. The booklet proved a useful tool for offering reliable information, which was more readily received as explained by one CHW below:

‘I am talking about TB, this is the booklet. What I am saying is written here so that they [*patients and community members*] can agree. Sometimes they don’t trust us as CHWs, they want to see the real[*ity*].’ (Participant 19, female, SSI 4)

### Community health workers’ opinion of the booklet

Community health workers described the booklet as simple and easy to understand for them as well as patients, who enjoyed it so much, that they wanted their own copy.

‘They got a good understanding of what is happening there…. They said, “we going to keep this [*booklet*]”. It’s quite interesting for me because they want to show their children too because the time when we were around [*at the home visit*] the children was in school.’ (Participant 12, female, FGD 3)

A CHW in charge of a child support group mentioned that the pictograms made it easier for children to comprehend the information.

‘I use this in a children support group and it’s easy for them to understand because there are pictures.’ (Participant 16, female, FGD 3)

The availability of the booklet in different languages further improved understanding of information:

‘… If a person can’t read English you can give them the Xhosa booklet so they can … know what we are actually saying to them.’ (Participant 16, female, FGD 3)

With the improvement in patient counselling, CHWs were excited about and enjoyed using the booklet. They appreciated the fact that it had been designed specifically for them, taking into account their needs and opinions, and they felt that it inspired them in their role as educators. They also commented that they would like to continue using information materials in their practice.

‘You have … actually inspired us in a way we haven’t expected and we would actually like to thank you for coming up with something like this … and we would like to keep it going not just for now.’ (Participant 11, female, SSI 1)

### Suggested broader application for similar information materials

Community health workers were adamant that patients would benefit from similarly designed information materials, as it would prompt patients to read the information and increase their knowledge and awareness about TB. They also identified others in community-based settings based on their interactions who would benefit from similar simple, illustrated TB information materials, including teachers in schools, carers in crèches and carers in old-age homes, as it would serve as a tool in informing and educating these groups.

‘It can be useful if you are going to schools. You can leave this booklet with the teachers so the teachers can read it and they can use it.’ (Participant 7, female, FGD 2)

Community health workers suggested that posters with selected information be placed in the patient waiting areas of clinics. This was advocated as a good idea to use as a reference source to assist in patient education, particularly for those patients who missed the health education talks.

‘When we educate the client about TB you need to show the client that there is a poster. If you are still waiting [*to see a health care professional*], then you can just go around to look while we are doing education.’ (Participant 1, female, FGD 1)

## Discussion

This is the first reported study that has developed TB materials specifically for CHW use and assessed its impact on knowledge as well as perceptions of its usefulness in patient interactions. A simple, illustrated booklet tailored for use by CHW acted as a tool for personal knowledge acquisition as well as a patient communication and education tool. The booklet improved knowledge significantly, enhanced confidence in answering questions and facilitated communication with patients, supporting previous findings.^17,18,23^ However, a key distinguishing aspect is that the information materials were not linked to a lengthy training process or to supervision, but included only a short 20 minute information session with CHWs.

Community health workers must have good knowledge to facilitate case finding and appropriate case management, the two foundations of TB prevention and control.^38^ Baseline knowledge in this study was 70.6%, which is higher than that reported in other studies,^39,40^ although a direct comparison is limited because of the different measures employed to assess knowledge. Misconceptions relating to the cause of TB, such as smoking and cold weather, were concerning given the integral role that these front-line workers play in communicating information about the disease throughout the community. If these incorrect perceptions mirror those within the local community, they could be a reason for delayed health care seeking.^39^ After use of the booklet, knowledge of the cause of TB significantly improved from 54% to 93%.

It was concerning that, in a health care system often overwhelmed by patients with multiple conditions, CHWs were unaware of the valuable medicine-related advice they could offer to patients, for example co-administration with other medicines. Their knowledge regarding side effects was similarly poor. Patients with TB want side effect information as they feel it improves their understanding of the medication and, if aware of what to expect, can reduce the possibility of defaulting.^41^ Given that TB health care delivery in South Africa is moving towards task shifting to CHWs through the primary health care re-engineering strategy, poor TB treatment-related knowledge may negatively affect the ability of CHWs to fulfil their roles as patient educators and adherence supporters.

The two areas of low baseline knowledge in drug-resistant TB were the meaning of XDR-TB (36%) and its treatment duration (55%). Given the increasing incidence of drug-resistant TB,^2^ inadequate CHW knowledge can have severe consequences if, for example, CHWs fail to identify such patients and fail to refer the patient to the higher levels of health care. As such, XDR-TB knowledge, which only increased to 39% at follow-up, and treatment duration, which increased to 74%, should be highlighted as key areas for training and intervention.

The success of the booklet in appealing to CHWs is partly because of their central involvement in the design process, stimulating ownership of the final product and, as noted by others, ensuring the relevance of the intervention in their practice.^42^ The other key factors in increasing booklet appeal were the simple text and the pictograms, the latter reportedly resulting in enhanced recall of TB information, as has been previously found.^18^ The reported success of the pictograms could be attributed to the attention paid to their design and development to ensure cultural relevance and visual simplicity.^20,21,43^ Although the use of simple text has been advocated mainly for the design of patient information materials,^43^ study CHWs were highly appreciative of the simplicity of the text, supporting previous findings for designing information materials for HCPs.^34^ The availability of the booklet in three different local languages increased acceptability among both CHWs and patients and is a strategy for improving understanding for limited literacy individuals.^21^

Health care professionals, including CHWs, need to be adequately trained to improve their communication skills when interacting with patients.^10,18^ This is particularly important in counselling patients with TB where the quality of the interaction between patients and HCPs has been linked to adherence.^10^ The booklet had a dual positive effect on CHW practice as it improved their interpersonal relationship with patients as a result of improved communication within the dyad, and they found the patient education process easier and more exciting. This highlights the potential value of having a well-designed educational tool to improve CHW practice. In addition, the use of the booklet by the CHWs facilitated their own work-based learning, which contributed to building their capacity as well as improving learning for patients and community members. Although HCPs often cite the lack of time as a barrier to using patient education tools during consultations,^13^ CHWs in this study found that the inclusion of pictograms made it easier to explain certain concepts, which resulted in saved time. Community health workers tend to have fewer time constraints than other HCPs such as nurses or doctors and so can communicate with the patient in a more relaxed manner.

Research has found that the community may perceive the CHWs’ knowledge, skills and overall abilities as being inferior, leading to general distrust.^42^ Study CHWs commented on the lack of patient trust displayed for verbally communicated information. For health information to be accepted and internalised by patients, it needs to be adequate, trustworthy and easily comprehended.^44^ The availability of simple written information materials to validate verbal information can therefore be useful in overcoming this trust barrier and creating a more receptive environment for education. Study CHWs reported better patient understanding about the disease and greater participation during interactions.

Limitations include the limited generalisability of the quantitative findings, as CHWs were drawn from one district only. Despite recruiting all available local CHWs, the sample size was low which may have reduced the possibility of identifying significant relationships within the data. Future research could involve a follow-up large-scale intervention study to formally assess the impact of illustrated, simple materials on CHW practice. An additional focus on eliciting patient opinion within such a study would allow the voice of these often disempowered patients to be heard.

## Conclusion

This study found that the use of a user-friendly, simple, pictorial-based booklet can improve CHW knowledge and contribute to enhanced confidence in their role as a CHW by facilitating better communication with patients and improving patient trust. Collaboration with the CHWs during the design process, careful consideration of booklet content and format, and the inclusion of well-designed pictograms emerged as factors influencing its ease of use, and therefore its acceptability. The booklet had a dual effect of improving their interpersonal relationship with patients because of improved communication within the dyad and of allowing for a more exciting and less difficult patient education process.
